# Development of a Sensitive Enzyme-Linked Immunosorbent Assay and Rapid Gold Nanoparticle Immunochromatographic Strip for Detecting Citrinin in *Monascus* Fermented Food

**DOI:** 10.3390/toxins10090354

**Published:** 2018-09-02

**Authors:** Shih-Wei Wu, Yao-An Yu, Biing-Hui Liu, Feng-Yih Yu

**Affiliations:** 1 Graduate Institute of Medicine, Chung Shan Medical University, Taichung 402, Taiwan; shawn1024200288@gmail.com; 2 Department of Biomedical Sciences, Chung Shan Medical University, Taichung 402, Taiwan; maoliu1966@gmail.com; 3 Graduate Institute of Toxicology, College of Medicine, National Taiwan University, Taipei 10051, Taiwan; 4 Department of Medical Research, Chung Shan Medical University Hospital, Taichung 402, Taiwan

**Keywords:** Citrinin, ELISA, gold nanoparticle immunochromatographic strip, *Monascus* fermented food

## Abstract

Antibodies against citrinin (CTN) were generated from rabbits, which were injected with CTN-keyhole limpet hemocyanin (KLH). This work involved the development of a sensitive competitive direct enzyme-linked immunosorbent assay (cdELISA) and a rapid gold nanoparticle immunochromatographic strip (immunostrip) method for analyzing CTN in *Monascus*-fermented food. CTN at a concentration of 5.0 ng/mL caused 50% inhibition (IC_50_) of CTN-horseradish peroxidase (CTN-HRP) binding to the antibodies in the cdELISA. The capable on-site detection of CTN was accomplished by a rapid antibody-gold nanoparticle immunostrip with a detection limit of 20 ng/mL and that was completed within 15 min. A close inspection of 19 *Monascus*-fermented foods by cdELISA confirmed that 14 were contaminated with citrinin at levels from 28.6–9454 ng/g. Further analysis with the immunostrip is consistent with those results obtained using cdELISA. Both means are sensitive enough for the rapid examination of CTN in *Monascus*-fermented food products.

## 1. Introduction

Citrinin (CTN) is a secondary metabolite synthesized from *Aspergillus* and *Penicillium* and contaminates corn, wheat, barley and rice [1,2]. CTN is also a secondary metabolite that is synthesized by *Monascus* spp. (red yeast), which is a fungal strain that is traditionally used in brewing rice. *Monascus*-fermented products are widely added to meat and wine as a food additive pigment. In western countries, fermentation products developed by *Monascus* are considered health food and are widely used to prevent cardiovascular diseases [3]. Recently, the Taiwan Food and Drug Administration (Taiwan-FDA) reported that 58 of 84 commercially available *Monascus*-fermented rice samples collected in 2009–2012 were contaminated with 0.4–93.5 ppm of citrinin, and the contamination of citrinin in fermented rice is very serious [4]. Therefore, the toxicity and content of CTN in *Monascus* health foods has become a public concern. In 1986, the IARC (International Agency for Research on Cancer) categorized CTN as a group 3 carcinogen but with insufficient evidence of its carcinogenicity [5]. In Taiwan, the maximum allowable level of CTN in red yeast rice, *Monascus* products, and *Monascus* colors are 5 ppm, 2 ppm and 0.2 ppm, respectively. Japan set a limit of 0.2 ppm CTN in red fermented products [4].

Currently, high-performance liquid chromatography with fluorescence detection (HPLC-FL) and liquid chromatography with tandem mass spectrometry (LC/MS/MS) are used to measure CTN levels in food [6,7,8,9,10]. The advantages of HPLC and LC/MS/MS methods are their high accuracy and reproducibility, but they have drawbacks, for example, they require expensive equipment, qualified personnel, and time-consuming sample preparation. Thus, analyzing large amounts of samples in a short time using these methods is impossible. Therefore, rapid, easy-to-use, immunochemical methods for detecting CTN in fermented foods have been developed. [11,12,13,14]. However, most immunochemical methods cannot be used for on-site testing attributable to their multiplex washing steps, long reaction time and the need for expensive instrumentation. Hence, on-site detection methods that are rapid, stable, reliable and affordable must be developed.

The advantages of gold nanoparticles are their high stability and high absorption coefficient. Gold nanoparticles can be synthesized promptly and can be conjugated easily with an antibody [15,16]. Therefore, gold nanoparticles are widely used in biosensor and immunochromatographic strips (immunostrip) for detecting mycotoxins [17,18]. In an immunostrip, the test samples and gold nanoparticle-antibody conjugates migrate by capillary action onto a nitrocellulose membrane where they interact with the test and the control line. The colored gold nanoparticle-antibody conjugates offer a quick on-site testing in less than 15 min without additional equipment and provide visible results [19,20]. Previous reports have shown that many samples from diverse regions are contaminated with various levels of CTN [3,21,22,23]. In this investigation, a specific polyclonal antibody against CTN was produced and successfully used in a sensitive ELISA and a gold nanoparticle immunostrip for analyzing CTN in *Monascus*-fermented food.

## 2. Results

### 2.1. Production and Characterization of Antibodies

**Figure 1:**
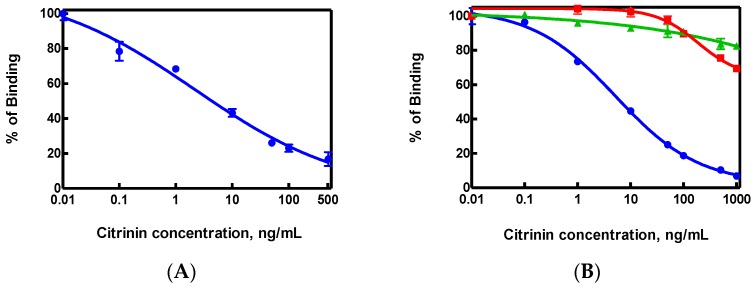
(**A**). The standard curve of citrinin (●) in a ciELISA. (**B**). Cross-reactivity of the CTN polyclonal antibody with CTN (●), ochratoxin A (■), and 1-hydroxy-2-naphthoic acid (▲) as determined by a cdELISA. Data were calculated by the average of three sets of experiments. The absorbance of the control, A0, with no toxin present was 1.5.

New Zealand rabbits received four immunizations with CTN-KLH conjugates as immunogens that would cause them to generate antibodies for citrinin. Both competitive indirect and direct ELISAs were then utilized to ascertain whether the antisera yielded antibodies specific to citrinin. The antiserum titer reached its highest level in the 70th week. Therefore, antiserum from the 70th week was conducted in the following study. The concentration that caused 50% inhibition of the binding of antibodies to the solid-phase citrinin—ovalbumin (CTN-OVA) by free citrinin (IC_50_) was 4.6 ng/mL in the ciELISA (Figure 1A). The concentration that caused 50% inhibition of the binding of CTN-HRP to the antibodies by citrinin (IC_50_) was calculated as 5.0 ng/mL in the cdELISA. The detection limit of citrinin (IC_10_) was calculated to be 0.2 ng/mL (Figure 1B) in accordance of a 90% confidence interval at 10% of inhibition of binding of the CTN-HRP conjugate. This cdELISA was further used to test whether the antibodies exhibited cross-reactivities with other mycotoxins. According to Figure 1B, mycotoxins, for example, ochratoxin A (OTA) and 1-hydroxy-2-naphthoic acid whose structures are similar to that of citrinin, showed weak cross-reactivity with the antibodies. The concentration that caused 50% inhibition of the binding of antibodies to the CTN-HRP by 1-hydroxy-2-naphthoic acid and OTA exceeded 1000 ng/mL (Figure 1B). Other mycotoxins, including patulin, zearalenone, aflatoxin and deoxynivalenol at a concentration 1000 ng/mL, could not inhibit the antibodies bind to the CTN-HRP conjugate in the cdELISA (data not shown).

### 2.2. Analytical Recovery of CTN Spiked to Red Yeast Rice Samples by cdELISA

A recovery study was conducted to examine the cdELISA correctness for the analysis of CTN which was spiked in red yeast rice samples. Results from the cdELISA are presented in Table 1. When the CTN at spiked levels of 100 to 500 ng/g, the analytical recovery rates were found to be 81–86% with coefficient of variation (CV) values less than 5%. On the other hand, the recovery rate and CV value of the sample spiked with 5000 ng/g CTN was 76% and 11.6, respectively; this sample was subjected to 5 fold more dilution for cdELISA in order to lie within the linear portion of standard curve. The overall average of analytical recovery for all the 100–5000 ng/g samples was found to be 85% (CV, 7.65). These data suggested that the cdELISA is a feasible method for detecting CTN in red yeast rice samples.

### 2.3. Assay of CTN in the Samples Using cdELISA

The effectiveness of cdELISA for measuring citrinin levels in fermented samples was assessed by collecting 19 brand-name samples and one control sample (rice) from local markets and testing them using cdELISA. To measure the level of citrinin more accurately and to prevent interference that would otherwise be caused by the extraction solution, the sample extractions were diluted 10-fold with 0.01 M PBS buffer and analyzed by the cdELISA. Samples 1 to 6 all contained high concentrations of citrinin from 1628 to 9454 ng/g (1.628 to 9.454 µg/g) (Table 2.). Moreover, the concentrations in sample 5 and 6 both exceeded the Taiwan-FDA regulatory limit of 5 µg/g (ppm) for citrinin in red yeast rice. Samples 7 to 19 from other *Monascus*-fermented food, there are five not detected samples, the others contained citrinin at levels from 28.6 to 64.4 ng/g, which did not exceed the 2 µg/g (ppm) of Taiwan-FDA regulatory limit for citrinin in *Monascus*-fermented products.

### 2.4. Fabrication of the Immunostrip

**Figure 2:**
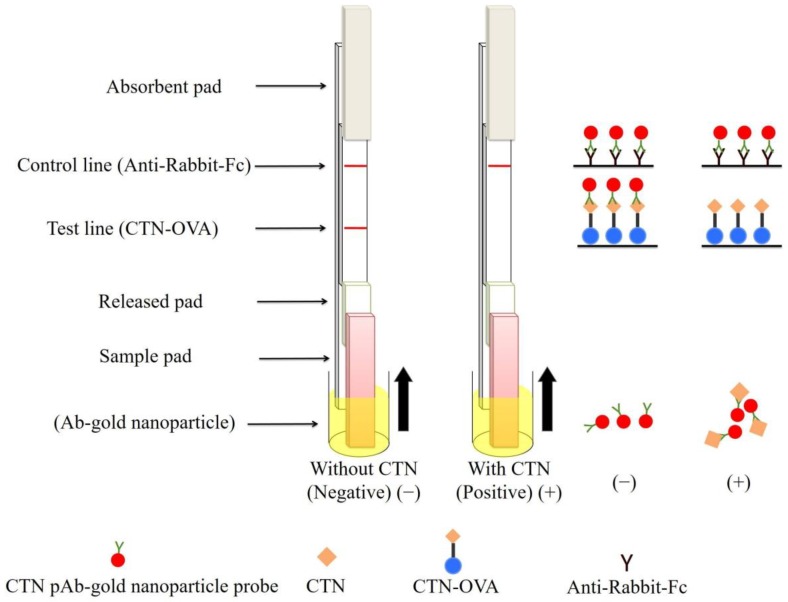
A graphic description of the immunostrip. Ab-gold nanoparticle; C, control line (Goat anti-rabbit IgG); T, test line (CTN-OVA); released pad.

The immunostrip provides a suitable means of detecting citrinin in *Monascus*-fermented products on-site and is rapid and does not involve cumbersome operations. When the citrinin level in the sample surpasses a particular value, the toxin captures all of the binding sites of the antibody-gold conjugates in the sample solution or in the “conjugated pad”. Therefore, no available antibody-gold conjugate can bind with CTN-OVA in the test zone, and no red line is visible in the test zone, which indicated a positive result. The correctness of the assay is verified using a control zone with a goat-anti-rabbit secondary antibody. With proper operation, a red line should always be visible in the control zone in spite of the presence or absence of citrinin. Therefore, the testing of a citrinin-negative sample should always produce two red lines on the membrane, whereas a toxin-positive sample should only display one (Figure 2).

### 2.5. Visual Detection Limit of an Immunostrip for CTN

**Figure 3:**
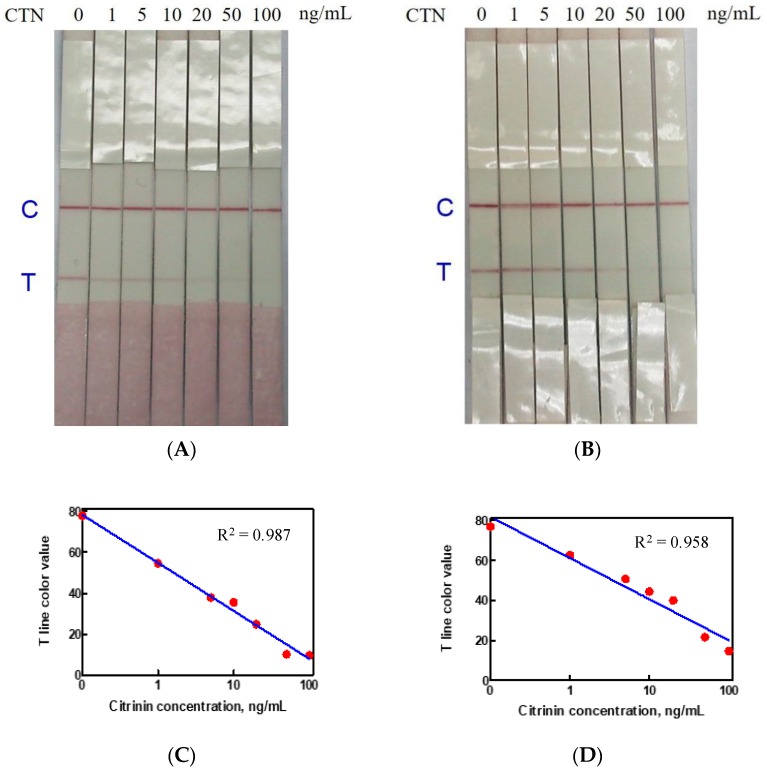
The visual detection limit of the immunostrip for CTN. Different concentrations (0–100 ng/mL) of a CTN certified standard was dissolved in PBS. (**A**) The visual detection limit is 20 ng/mL using 8 μL of Ab-gold nanoparticle conjugates were loaded into the sample solution; (**B**) the visual detection limit is approximately 20~50 ng/mL when 8 µL of conjugates were absorbed on the release pad. (**C**) The standard curve of the T line color density value without the release pad; when the value is less than 25 it indicates a positive result. (**D**) The standard curve for the T line color density value with the release pad; when the value is less than 25 it indicates a positive result. Each concentration was tested three repeats.

Citrinin certified standard (0–100 ng/mL) were subjected to immunostrip testing where CTN-OVA and goat anti-rabbit were drawn on the test zone and control line, respectively. When the citrinin certified analytical standard solution and the antibody-gold nanoparticles conjugates were directly mixed in a microplate well and the immunostrip was dipped into the well, the detection limit of the immunostrip was 20 ng/mL (Figure 3A). When the citrinin level exceeded 20 ng/mL, the citrinin completely occupied the binding sites of the conjugates then no available antibody-gold nanoparticles could bind to the CTN-OVA in the test zone. Therefore, the immunostrip exhibited only one red line in the control zone. The color density on the test line was also analyzed by a strip scan reader and this established the standard curves, whereas the T line color density value is less than 25, which indicated a positive result (Figure 3C). Alternatively, when the antibody-gold nanoparticles were absorbed by the release pad, the visual detection limit of the immunostrip was 20–50 ng/mL (Figure 3B) while the T line color density value was less than 25 (Figure 3D). Accordingly, the samples and antibody-coated gold nanoparticles were mixed first and then subject to the immunostrip for sample analysis.

### 2.6. Assay of CTN in Samples with the Immunostrip

**Figure 4:**
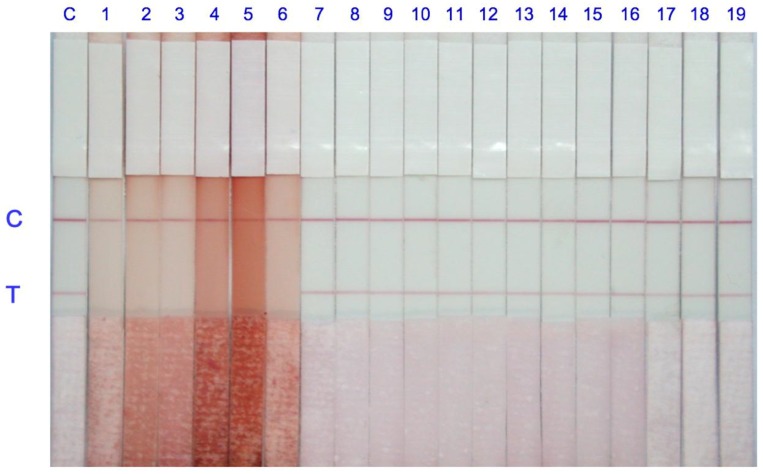
Analysis of CTN with the immunostrip in 19 red yeast fermented samples and one control rice sample. Samples 1–6 showed that the red line vanished in the test zone, which verified that they are CTN positive. Samples 7–19 containing CTN less than 20 ng/mL displayed two red lines indicating that they are negative. Each sample was tested three repeats.

The 19 extracted red yeast fermented samples and one control sample (rice) were added to the microplate well to determine citrinin contamination using immunostrips. According to Table 2, samples 1 to 6 contained citrinin levels that were much higher than 20 ng/mL. Samples 7 to 19 contained citrinin levels lower than 20 ng/mL in the extraction solution. Therefore, in Figure 4, sample 1 to 6 yielded positive results with only a reddish line in the control zone of the membrane. Further dilutions of samples 1–6 (1:10; 1:20; 1:50) were subjected to the immunostrip test. The results were presented in the Figure S1. All dilution samples examined with immunostrips were positive with only a reddish line in the control zone which are consistent with the cdELISA results. Samples 7 to 19 yielded negative results with one clear red line in the test zone and the other in the control zone of the immunostrip.

### 2.7. Comparison of Gold Nanoparticles Size for Immunostrip

**Figure 5:**
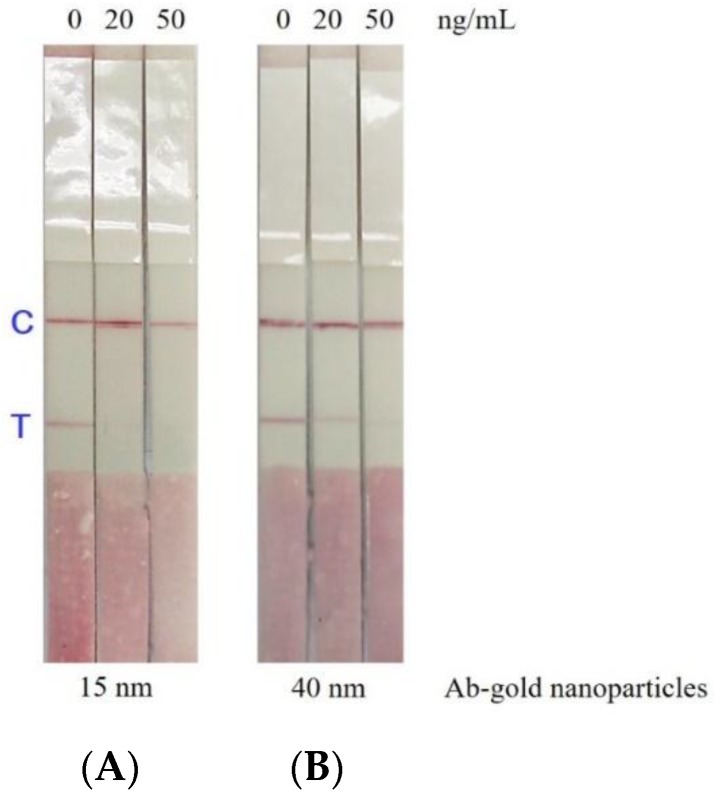
Two different sizes of gold nanoparticles were used for conjugation with antibodies. The competitiveness of the antigen on the test line with antibody-gold nanoparticles of 15 nm (**A**) is better than that of the 40 nm particles (**B**).

To test whether the size of the gold nanoparticles will affect the sensitivity of the immunostrip or not. Two different size of gold nanoparticles with diameters of approximately 15 or 40 nm were synthesized and conjugated with antibodies specific for CTN. Figure 5 shows that the results using antibody-gold nanoparticles of 15 nm (Figure 5A) are better than that of using antibody-gold nanoparticles of 40 nm (Figure 5B) based on a T line color density by the strip scan reader.

## 3. Discussion

Food that is contaminated with mycotoxins such as citrinin affects human health [24]. Thus, the development of a quick detection means for citrinin is very important, simplifies the preparation of samples and reduces the cost of analysis of the critical points. This study focuses on the ELISA and the development of an immunostrip. Both methods are simple and more rapid than that of the chemical methods. CTN is small molecular weight mycotoxin, which is non-immunogenic but has to be coupled with a protein carrier to exhibit this immunogenicity. Although CTN has a carboxylic acid group that could be conjugated to a protein carrier using a water-soluble carbodiimide method, the hydroxyl group on the eighth carbon forms a hydrogen bond with carboxylic acid on the 14th carbon, and the ketone group on the sixth carbon forms a hydrogen bond with carboxylic acid on the 14th carbon [12,25]. These hydrogen bonds eliminate the capacity of the carboxylic acid to conjugate with a protein carrier with an amine group. Therefore, the carbodiimide method is not suitable for conjugating CTN with proteins. CTN also has a methyl group that could be conjugated to a protein carrier by the Mannich method using formaldehyde [26], and therefore, a formaldehyde method was used herein to conjugate CTN with keyhole limpet hemocyanin (KLH) to make it a valid antigen to produce specific antibodies for citrinin.

The high sensitivity of cdELISA and ciELISA depends on the quality of the CTN-HRP conjugates and CTN-OVA conjugates. In this study, the conjugation of citrinin and HRP with a molecular ratio 44:1 with the Mannich method maximized the cdELISA sensitivity, and the conjugation of citrinin and OVA with the molecular ratio 22:1 by the Mannich method maximized the ciELISA sensitivity. In the ciELISA, the concentration that caused 50% inhibition of the binding of antibodies to the solid-phase CTN-OVA by free citrinin (IC_50_) was calculated to be 4.6 ng/mL (Figure 1A). In cdELISA, the concentration that caused 50% inhibition of the binding of CTN-HRP to the antibodies by CTN (IC_50_) was 5.0 ng/mL (Figure 1B). Ochratoxin A and synthetic compound 1-hydroxy-2-naphthoic acid whose portion of structures are similar to that of citrinin, showed 10% weak cross-reactivity with the antibodies at a level of 100 ng/mL. Although Kong, Xie, Liu, Song and Kuang (2017) [27] reported a ciELISA and immunostrip for CTN, they had not developed the cdELISA method and were also short of the real sample analysis. Due to the cdELISA is more simple and rapid than ciELISA, this study devoted to the development of cdELISA and its application to screening a large amount of red yeast fermented samples. In cdELISA, the samples were extracted using 100% methanol and were generally diluted at least 10-fold with 0.01 M PBS to avoid solvent and matrix interference. The cdELISA can tolerate up to 10% methanol in the analyzed samples [28] and its detection limit is determined to be 0.2 ng/mL in accordance with a 90% confidence interval at 10% of inhibition of binding of the CTN-HRP conjugate. (Figure 1B).

The Taiwan FDA has set regulatory limits of 200 ppb, 2 ppm and 5 ppm on citrinin in red yeast rice pigments, red yeast food and red yeast rice, respectively. Japan also set a regulatory limit of 200 ppb in red yeast pigment [4]. The results of this study reveal that two out of six red yeast rice samples that were collected in a local supermarket contained more than 5 ppm of citrinin and one contained 9.5 ppm, which is almost two times that of Taiwan’s regulatory limits (Table 2). However, some other red yeast samples that were contained with CTN levels that did not violate the limit. This article involved in using immunochemical method such as ELISA and rapid gold nanoparticle immunostrip for rapid and on-site detection of citrinin. In order to confirm the efficacy and correctness of sample analysis by using cdELISA, we have analyzed the recovery of CTN spiked to red yeast rice samples. The results indicated that the recovery rates were found to be 76–97% which suggested that the cdELISA is a feasible method for detecting CTN in the samples. Chemical analytical method such as HPLC for citrinin analysis is not our main focus. Detecting citrinin in red yeast rice samples using HPLC was carried out and published by Taiwan Food and Drug Administration. Liao, et al. [4] have reported that 58 of 84 commercially available *Monascus*-fermented rice samples collected in 2009–2012 were contaminated with 0.4–93.5 ppm of citrinin. Therefore, the contamination of citrinin in fermented rice is very serious.

There are several advantages of the immunostrip over ELISA include time-savings and the ease of application by the user. Unskilled personnel without using any devices can acquire analytical results using the immunostrip in 15 min, which makes this method very feasible for the on-site testing of CTN. This is the first work to develop an immunostrip for detecting CTN in red yeast fermented samples. Even though large amounts of antibody and CTN-OVA conjugates are required for each sample assay, compared to the ELISA, which results in a slightly higher cost than the ELISA, the obvious advantages warrant its further development. Moreover, the absence of an enzyme reaction in the immunostrip provide its stability during a longer-term storage. Figure 3A shows that the visual detection limit of the immunostrip is 20 ng/mL, which is below the regulatory limits of Taiwan. So that our immunostrip can meet the regulatory limit test requirements, we set the different dilution factors of 10, 100 and 250 for yeast rice pigments, red yeast food and red yeast rice, respectively. With respect to sample analysis, the results in Figure 4 are consistent with the data in Table 1, which proves that this immunostrip can be used to detect CTN in red yeast samples. According to a recent report [29], most immunostrip researchers did not detect toxic bacteria or toxins from real food using their technologies. Many groups of researchers artificially added and spiked toxins before detection. There was a limit to the application. However, our study succeeded in detecting toxins from actual fermented foods using immunostrips. As previous studies have indicated, the intensity of the color and the competition between CTN in the sample and the CTN-OVA in the test line can be affected by the size of the antibody-gold nanoparticle conjugates [30,31]. Therefore, this study follows Frens (1973) [31] in the synthesizing of gold nanoparticles with diameters of approximately 15 or 40 nm and then conjugating these different sizes of gold nanoparticles with antibodies specific for CTN. Figure 5 indicates that the results using antibody-gold nanoparticles of 15 nm are better than that of using antibody-gold nanoparticles of 40 nm based on a T line color density by the strip scan reader. However, to develop a highly sensitive immunostrip, the decision was ultimately made to use the 15 nm gold nanoparticles to conjugate with antibodies specific to CTN and without a release pad.

## 4. Conclusions

This work developed antibodies against citrinin and is the first occurrence of their use in an immunostrip to detect CTN in red yeast samples. The concentration of CTN caused 50% inhibition of the binding of CTN-HRP to the antibodies and was calculated to be 5.0 ng/mL in the cdELISA, and the detection limit was determined to be 0.2 ng/mL. The immunostrip had a visual detection limit of 20 ng/mL in 15 min and it is sensitive enough for analyzing red yeast samples. The methods developed can have a vital role for the rapid detection of CTN in red yeast fermented food.

## 5. Materials and Methods 

Citrinin, certified analytical standard, ochratoxin A, 1-Hydroxy-2-naphthoic acid, ovalbumin (OVA), bovine serum albumin (BSA), Keyhole limpet hemocyanin (KLH), Tween 20, Hydrogen tetrachloroaurate (III), and trihydrate (HAuCl4) were obtained from Sigma (St. Louis, MO, USA). Ammonium sulfate and sodium acetate were purchased from J. T. Baker (Phillipsburg, NJ, USA). Methanol and formaldehyde were purchased from Merck (Darmstadt, Germany). Goat anti-rabbit IgG-HRP and Horseradish peroxidase (HRP) were obtained purchased from Roche (Mannheim, Germany). Microplates, Freund’s complete adjuvant, Freund’s incomplete adjuvant and goat anti-rabbit IgG were purchased from Thermo (East Grinstead, UK). HRP substrate solution 3,3′,5,5′-tetramethylbenzidine (TMB) was purchased from Neogen Corp (Lexington, KY, USA). An Easypack kit was purchased from MDI Membrane Technologies (Ambala, India). The Double Axes Programmable Controller (model P602C) for drawing the test line and the control line of the membrane was purchased from Troy (Taichung, Taiwan). The 0.22 μm syringe filter was provided from Gelman Science (Ann Arbor, MI, USA). All of the organic solvents and other chemicals used were reagent grade or better. The New Zealand Rabbits (8 weeks old, female) were ordered from Da Zong Farm (Changhua, Taiwan). A Vmax ELISA reader was obtained from Molecular Devices Co. (Menlo Park, CA, USA). The immunostrip scan reader was obtained from Taiwan Advance Bio-Pharmaceutical Inc. (Taipei, Taiwan).

### 5.1. Preparation of the Different CTN Conjugates

#### 5.1.1. Coupling of CTN to KLH

CTN was covalent coupled to KLH with formaldehyde to serve as an immunogen for immunizing the rabbits [26]. Briefly, the KLH solution (5 mg of KLH in 0.5 mL of 0.1 mM sodium acetate solution, pH 4.2) was added to CTN (1 mg) in 1 mL methanol with constant stirring at room temperature. Then, 0.32 mL of a 36.5% formaldehyde solution was added to the mixture with steady stirring at room temperature for 72 h and stirred at 4 °C for another 16 h. Finally, the mixture was dialyzed against 0.01 M PBS for 72 h with four exchanges of 0.01 M PBS. The products were stored at −20 °C until used.

#### 5.1.2. Conjugation of CTN to OVA

As a coating antigen for ciELISA and a control line for the immunostrip, the CTN was conjugated to OVA using formaldehyde [26]. Generally, 8 mg of OVA in 1.28 mL 0.1 mM sodium acetate solution (pH 4.2) is added to CTN (1 mg) in 1 mL of methanol with constant stirring at room temperature. Next, 0.32 mL of the 36.5% formaldehyde solution was added gradually to the OVA combination with steady stirring at room temperature for 72 h and then stirred at 4 °C for another 16 h. Following the coupling reaction, the combination was dialyzed in 0.01 M PBS with the buffer changed 3 times within 72 h and then stored at −20 °C until used.

#### 5.1.3. Conjugation of CTN to HRP 

Coupling of CTN to HRP was also completed using formaldehyde [26]. Generally, the HRP solution (0.8 mg of HRP in a 0.8 mL of 0.1 M sodium acetate solution, pH 4.2) was added to CTN (0.2 mg) in 0.1 mL methanol with steady stirring at room temperature. Afterward, 0.04 mL of a 36.5% formaldehyde solution was added slowly to the HRP solution with steady stirring at room temperature for 72 h. After the room temperature reaction, the mixture was stirred at 4 °C for 16 h and dialyzed against 1 L of 0.01 M PBS for 48 h with five buffer exchanges. After dialysis, the reactant was kept for the cdELISA analysis.

### 5.2. Generation of Polyclonal Antibody

The immunization method was similar to those previously reported [28]. New Zealand rabbits (8 weeks old and female) were immunized with 0.5 mg of CTN-KLH in 0.5 mL of 0.01 M PBS that had been emulsified with an equal volume of Freund’s complete adjuvant by intradermal injection to generate the polyclonal antibody against CTN. After an initial injection for four weeks, the booster antigen containing 0.5 mg of CTN-KLH in 0.5 mL of PBS and the 0.5 mL of Freund’s incomplete adjuvant was subcutaneously injected at 4 sites on the thigh. The antiserum was collected from the ears of the rabbit beginning at the sixth week. The antiserum was precipitated 2 times with 35% (NH_4_)_2_SO_4_. The final precipitate in (NH_4_)_2_SO_4_ was dissolved in distilled water the same as half of the original volume. Afterwards, the mixture was dialyzed in 1 L of 0.01 M PBS and the PBS buffer was changed 3 times within 24 h at 4 °C.

### 5.3. ciELISA

The procedure of ciELISA was carried out based on the method primarily described [19,32]. CTN-OVA (0.1 mL and 2 µg/mL) was coated onto each well of a plate and kept at 37 °C for 1 h. After 1 h of incubation, the plate was washed four times with PBS-Tween (0.35 mL per well; 0.05% Tween 20 in 0.01 M PBS). Then, 0.2 mL of PBS-BSA (0.2 mL per well; 0.1% BSA in 0.01 M PBS) was added and allowed to incubate at 37 °C for 30 min. The plate was washed again after incubation, followed by adding a CTN standard to each well with a concentration from 0−500 ng/mL and 0.05 mL anti-CTN polyclonal antibody (5 μg/mL in 0.01 M PBS) for reaction at 37 °C for 1 h. After the plate was washed four times with PBS-Tween, 0.1 mL of the goat anti-rabbit IgG-HRP conjugate (1:20,000 dilution) was added and reacted at 37 °C for 1 h. The plate was washed and 0.1 mL of TMB substrate solution was added and the color development was permitted to occur for 15 min. The reaction was terminated by adding 0.1 mL of 1 N HCl. A ELISA reader (Vmax, Molecular Devices Co., Menlo Park, CA, USA) was used to read the 450 nm absorbance value.

### 5.4. cdELISA

The procedure for the cdELISA was the same as that previously reported [32]. The anti-CTN polyclonal antibody was coated to the plate (0.1 mL of 10 μg/mL in 0.01 M PBS and kept at 37 °C for 1 h. The plate was washed with PBS-Tween followed by blocking with PBS-BSA at 37 °C for 30 min. After these steps, the CTN standards (0−1000 ng/mL; 0.05 mL per well in 0.01 M PBS) or CTN samples (0.05 mL) together with the CTN-HRP conjugate (0.05 mL in 0.01 M PBS) were added and reacted at 37 °C for 1 h. The plate was washed again and then 0.1 mL of TMB substrate solution was added. After reaction at room temperature in the dark for 10 min, the reaction was stopped by adding 0.1 mL of 1 N HCl. The absorbance value at 450 nm was measured by a ELISA reader.

### 5.5. Analytical Recovery of CTN Spiked to Red Yeast Rice Sample by cdELISA

The red yeast rice sample collected from the local market was examined to be CTN negative by cdELISA first, and then one gram of grounded sample was spiked with CTN at concentrations ranging from 100 to 5000 ng/g. A control sample with no toxin added served as the blank. The protocol for sample extraction was similar to that previously described [4]. The spiked samples were kept in a cool room (4 °C) for 1 day and each sample in a 50 mL screwed capped bottle was added 10 mL of methanol for extraction. The samples were vortexed for 1 min and heated in the 70 °C water bath for 30 min. After cooling at room temperature, the supernatant of each sample was concentrated by a rotary evaporator and the final residue was reconstituted in 1 mL of methanol. The methanol solution was filtered through 0.22 μm nylon syringe filters and diluted with 9 mL of 0.01 M PBS for the following cdELISA analysis. Further dilution is needed if the sample concentration exceeds the highest point of the curve or the linear portion of standard curve.

### 5.6. cdELISA of Samples Contaminated with CTN

Nineteen samples, including red yeast rice, *Monascus* cookie and soy sauce samples, obtained from supermarkets were subjected to analyze the CTN levels. The protocol for sample extraction was similar to that above described [4]. Briefly, each sample (1 g) was extracted in 10 mL methanol and heated for 30 min at 70 °C with the water bath. Then, the supernatant was evaporated by a rotary evaporator and reconstituted in 1 mL of methanol. The methanol solution was filtered through 0.22 μm nylon syringe filters and diluted with 9 mL of 0.01 M PBS or more dilutions for both the cdELISA analysis and the immunostrip assay.

### 5.7. Construction of the Antibody-Gold Nanoparticle 

#### 5.7.1. Synthesis of Different Size Gold Nanoparticles

The process of synthesis was operated as the method previously published by Frens (1973) [31]. For the synthesis of 15 nm of gold nanoparticles, the 19.7 mg of HAuCl_4_ dissolved in 50 mL deionized water was heated with constant stirring. A condenser was adapted to avoid the evaporation of the solvent. Five mL of trisodium citrate (34 mM) was added while the solution was boiling. The color of the solution was changed in order (yellow, transparent, black and red) in 1 min, and the solution was cooled at room temperature. On the other hand, 2.5 mL of trisodium citrate (34 mM) was added to the 1 mM HAuCl_4_ solution. The resulting gold particle size was 40 nm. Transmission electron microscopy was used to confirm the diameter of the gold nanoparticles.

#### 5.7.2. Conjugation of the Antibody-Gold Nanoparticle. 

The process for the conjugation was related to that previously reported [19,33]. A titration result of 20 μg of CTN antibody dissolved in 0.1 mL of boric acid-borax buffer (pH 6.0) was added gradually to 2 mL of the gold nanoparticle (15 or 40 nm in diameter) solution with gentle stirring. The combination was reacted at room temperature for 30 min and stopped with 200 µL of filtered BSA for 30 min. After the reaction, the combination was centrifuged at 18,000× *g* for 30 min at 4 °C; the supernatant was then removed, and the antibody-gold pellets were reconstituted by adding 200 μL of 20 mM Tris-buffer saline (pH 8.0) with 1% BSA and 0.1% sodium azide. These CTN polyclonal antibody-gold nanoparticle were kept at 4 °C until use.

### 5.8. Preparation of the Immunostrip 

The assembly method of the immunostrip was achieved based on the method previously reported [19] with a slight modification. The immunostrip made up of three pads including sample, conjugate release and absorbent pads and the nitrocellulose membrane (NC membrane; pore size of 15 μm) with a test line and a control line. The test and control line of the NC membrane were drawn with 0.25 μL CTN-OVA (4 mg/mL) and 0.25 μL goat anti-rabbit IgG antibody (1 mg/mL), respectively, and then rested for 10 min at room temperature until the line was dried. Subsequently, the CTN polyclonal antibody-gold nanoparticle conjugate (8 μL per well) was applied to the sample solution or absorbed to conjugate release pad, which air dried at 37 °C for 10 min. The release pad was first pasted on the NC membrane by overcrossing 2 mm. Next, the sample pad was pasted on the release pad by overcrossing 2 mm. Finally, the absorbent pad was pasted on the topmost of the NC membrane for 2 mm crossover. The full assembled sheet was cut lengthways (4 mm) with an automatic cutter [34].

### 5.9. Assay of the CTN in Samples with the Immunostrip 

Analysis of the CTN in samples with the immunostrip, the antibody-gold nanoparticle conjugates (8 μL) were premixed with the sample solution (200 µL) or CTN standard solution. Each of the different concentrations of the CTN standard solution (0–100 ng/mL) or the sample solutions were loaded on the sample well. The immunostrips were dipped into the sample wells and the samples or CTN standard solution moved upward toward the membrane by capillary action. The results on the immunostrip were visual estimated after 15 minutes and the red line color density on the test line was analyzed by an immunostrip scan reader.

### 5.10. Data Analysis

The samples and standards were tested in triplicate and the mean values were obtained from the ELISA. Standard curves were established by plotting the absorbance value against the logarithm of the analyte concentration and fitted to a four-parameter logistic equation using GraphPad prism 5.0 software (La Jolla, CA, USA):

Y = {(A − D) ÷ (1 + (x/C) ^B^)} + D

where A is the asymptotic maximum (Absorbance value in the absence of an analyte, A_max_), B is the slope of the curve at the inflection point, C is the x value at the inflection point, and D is the asymptotic minimum (A_min_, background signal).

## Figures and Tables

**Table 1:** Analytical recovery of CTN spiked to red yeast rice samples by cdELISA.

Spiked CTN (ng/g) ^a^	ELISA (ng/mL)	ELISA (ng/g)	CV (%)	Recovery (%)
100	8.1 ± 0.4	81 ± 4	4.40	81
500	43.0 ± 1.4	430 ± 14	2.50	86
1000	96.5 ± 11.8	965 ± 118	12.10	97
5000	381.4 ± 44.8	3814 ± 448	11.60	76
Overal			7.65	85

**^a^** Each toxin level had two samples and each sample was run in triplicate.

**Table 2:** cdELISA and immunostrip analytical results of citrinin in red yeast fermented foods.

Samples	Food	ELISA (ng/mL) ^a^	ELISA (ng/g) ^a^	Immunostrip Assay
1	Red yeast rice	167.7 ± 4.1	1677 ± 41	+
2	Red yeast rice	468.3 ± 33.5	4683 ± 335	+
3	Red yeast rice	306.5 ± 24.7	3065 ± 247	+
4	Red yeast rice	162.8 ± 7.5	1628 ± 75	+
5	Red yeast rice	945.4 ± 80.6	9454 ± 806	+
6	Red yeast rice	698.7 ± 2.3	6987 ± 23	+
7	Cookie	ND ^b^	ND ^b^	−
8	Cookie	6.17 ± 0.84	61.7 ± 8.4	−
9	Cookie	ND ^b^	ND ^b^	−
10	Wine	4.21 ± 0.29	42.1 ± 2.9	−
11	Capsule	ND ^b^	ND ^b^	−
12	Bean	3.42 ± 0.13	34.2 ± 1.3	−
13	Soy sauce	2.86 ± 0.14	28.6 ± 1.4	−
14	Soy sauce	4.36 ± 0.11	43.6 ± 1.1	−
15	Sauce	ND ^b^	ND ^b^	−
16	Sauce	3.35 ± 0.1	33.5 ± 1.0	−
17	Sauce	4.93 ± 0.2	49.3 ± 2.0	−
18	Sauce	ND ^b^	ND ^b^	−
19	Sauce	6.44 ± 0.53	64.4 ± 5.3	−
Control	Rice ^c^	ND ^b^	ND ^b^	−

^a^ Each sample was extracted duplicate and analyzed in three repeats. ^b^ ND, not detected. ^c^ Rice is the control sample.

## Supplementary Materials

The following are available online at http://www.mdpi.com/2072-6651/10/9/354/s1, Figure S1: Various dilutions of extraction (1:10; 1:20; 1:50) of red yeast samples 1–6 were subjected to the immunostrip tests.
