# Supplementary material for: Development of a Sensitive Enzyme-Linked Immunosorbent Assay and Rapid Gold Nanoparticle Immunochromatographic Strip for Detecting Citrinin in Monascus Fermented Food
Source: Toxins (Basel). 2018 Sep 2;10(9):354. doi: 10.3390/toxins10090354 (PMC6162752; doi:10.3390/toxins10090354)
Supplement: Supplementary file 1 [file toxins-10-00354-s001.pdf]

Supplementary Materials: Development of a Sensitive Enzyme-Linked Immunosorbent Assay and Rapid Gold Nanoparticle Immunochromatographic Strip for Detecting Citrinin in *Monascus* Fermented Food

Shih-Wei Wu, Yao-An Yu, Biing-Hui Liu and Feng-Yih Yu

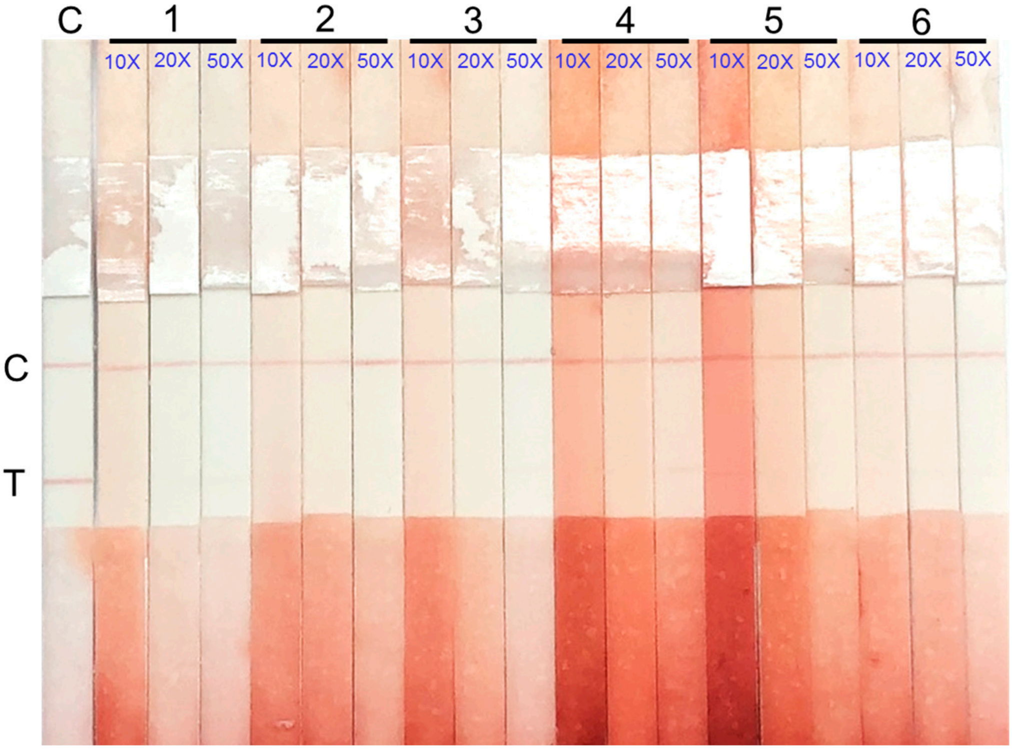

Figure S1. Various dilutions of extraction (1:10; 1:20; 1:50) of red yeast samples 1–6 were subjected to the immunostrip tests.
